# LICAVAL: combination therapy in acute and maintenance treatment of bipolar disorder

**DOI:** 10.1186/1745-6215-11-72

**Published:** 2010-06-23

**Authors:** Rodolfo N Campos, Luis F Costa, Danielle S Bio, Márcio G Soeiro de Souza, Carla RL Garcia, Frederico N Demétrio, Doris H Moreno, Ricardo A Moreno

**Affiliations:** 1 Department and Institute of Psychiatry, Clinical Hospital, University of Sao Paulo, School of Medicine, Brazil

## Abstract

**Background:**

The challenge of Bipolar Disorder (BD) treatment is due to the complexity of the disease. Current guidelines represent an effort to help clinicians in their everyday practice but still have limitations, specially concerning to long term treatment. LICAVAL (e*fficacy and tolerability of the combination of **LI**thium and **CA**rbamazepine compared to lithium and **VAL**proic acid in the treatment of young bipolar patients*) study aim to evaluate acute and maintenance phase of BD treatment with two combined drugs.

**Methods:**

LICAVAL is a single site, parallel group, randomized, outcome assessor blinded trial. BD I patients according to the DSM-IV-TR, in depressive, manic,/hypomanic or mixed episode, aged 18 to 35 years are eligible. After the diagnostic assessments, the patients are allocated for one of the groups of treatment (lithium + valproic acid or lithium + carbamazepine). Patients will be followed up for 8 weeks in phase I (acute treatment), 6 months in phase II (continuation treatment) and 12 months in phase III (maintenance treatment). Outcome assessors are blind to the treatment. The main outcome is the evaluation of changes in mean scores on CGI-BP-M between baseline and endpoint at the end of each phase of the study.

**Results:**

LICAVAL is currently in progress, with patients in phase I, II or III. It will extended until august 2012.

**Conclusions:**

Trials comparing specific treatments efficacy in BD (head to head) can show relevant information in clinical practice. Long term treatment is an issue of great important and should be evaluated carefully in more studies as long as BD is a chronic disease.

**Trial registration:**

ClinicalTrials.gov Identifier: NCT00976794

## Background

Bipolar Disorder (BD) treatment is a topic in evolution as long as the understanding of the clinical features, and possible pathophysiology, still progress. Due to multivariate factors associated with its cause and the variability of clinical presentations it's hard to determine a specific treatment with best outcome (efficacy and tolerability). Some difficulties in the BD treatment include: delay in diagnosis, high levels of comorbidity, frequent treatment nonadherence and high risk of relapse/recurrence (mainly in the presence of residual symptoms) [1].

Most treatments focus on acute phase and the measure of response as a reduction in symptoms of at least 50% from baseline. In fact, a number of patients who responded to treatment continue to experience significant subsyndromic symptoms. A small number of studies reported remission rates, which mean at least 2 months with no significant signs or symptoms of the disorder [2]. Fewer studies reported remission during acute phase through maintenance phase of treatment and its predictors [3] what is of great clinical significance. Recent data showed that BD treatment in special conditions was associated with full remission in only half of the patients and that nearly half of the recovered patients relapsed at least once during the two year of follow up [4]. Maintenance treatment is necessary in BD due to its great mortality, morbidity risk and social and professional impairment associated with its poorer outcome [5].

Lithium is first line choice for the maintenance treatment of BD, mainly for classic (euphoric) mania and bipolar depression according to many open controlled studies, with additional clinical effects such as: antisuicidal properties; augmentation and treatment of acute unipolar depression and recurrent depression [6]. The accumulating data tend to support its specificity in psychiatric usage, specially in those patients with "classic" BD. Available clinical trial suggest that better response to valproate are related to dysphoric or mixed episodes and rapid cycling patients [7]. Recently, valproate evidenced benefits on depressive aspects of BD both on acute and prophylactic use [8]. Carbamazepine is associated with better response in: not receiving treatment with mood stabilizers previously; atypical symptoms and signs; dysphoric and rapid cycling patients; treatment resistance to lithium therapy; under 30 years; no family history of BD [7]. Carbamazepine and valproate appear to be effective in the prophylactic treatment of bipolar disorder, especially in combination with lithium, although further studies are desirable [9].

In Brazil lithium is the first treatment choice in all BD phases representing an appropriate treatment of accessible cost. Treatment in the Brazilian Public Health System (*Sistema único de Saúde *- SUS) - have financial limitations once atypical antipsychotics and some anticonvulsants are not available for use in BD. Therefore, the most used treatments for the general population present smaller number of controlled studies in relation to the newest medications in which the pharmaceutical industry has interest in spreading. Although medications such as lithium, valproic acid and carbamazepine have been used for a long time, studies comparing effectiveness and other outcome measures, as well as data of long term combination treatment can still help the adaptation of the public health politics to the patient's real needs (Taveira and Moreno: Survey on treatment of bipolar disorder in Brazil: psychiatrists' epidemiology, prescription drugs and impact on heath policies. Submitted.)

## Methods

### LICAVAL Project

LICAVAL (*efficacy and tolerability of the combination of **LI**thium and **CA**rbamazepine compared to lithium and **VAL**proic acid in the treatment of young bipolar patients*) is a randomized trial designed to evaluate two combined drugs in the treatment of bipolar I patients.

### Design of LICAVAL

The key points of LICAVAL Project come from the rationale described above. We considered the disorder as a whole, that is, different clinical presentations (such as depression, mania or mixed episodes) are receiving the same treatment. The treatment focus is relapse/recurrence prevention and not treating a particular phase. Patients should have a long term follow up as BD is a chronic and recurrent disorder.

This a single site, parallel group, randomized, outcome assessor blinded trial. The study protocol was reviewed and approved by the appropriate institutional review board (Protocol number 0820/08) in accordance with the standards and guidelines established in the current amendment of the Declaration of Helsinki, and consistent with good clinical practice and applicable regulatory requirements. Written informed consent was obtained from all patients prior to any study-related activities. All phases of LICAVAL have their methodological details presented according to Consolidated Standard of Reporting Trials (CONSORT) 2010 Statement [10].

### Interventions

After the diagnostic assessments, the patients are allocated for one of the following groups of treatment:

Group I: lithium + valproic acid

Group II: lithium + carbamazepine

***Lithium***: Starting at 600 mg daily, dose weekly adjusted according to blood serum level (0,6 -1,2mEq/l), efficacy and tolerability

***Valproic acid***: Starting at 500 mg daily, dose weekly adjusted according to blood serum level (50 and 125 μg/ml), efficacy and tolerability

***Carbamazepine***: Starting at 200 mg daily and getting 600 mg daily at the end of the first week. Dose weekly adjusted according to blood serum level (8 and 12 μg/ml), efficacy and tolerability

Concomitant medications are permitted and may continue until remission or symptomatic control needed according to clinical criteria:

•*Lorazepam - 0,5 - 4 mg/day orally*

•*Sertraline - 50 - 200 mg/day orally*

### Eligibility

Patients are recruited in the Institute of Psychiatry, of the Clinical Hospital of the University of Sao Paulo, School of Medicine. BD I patients according to the DSM-IV-TR [2], in depressive, manic,/hypomanic or mixed episode, aged 18 to 35 years are eligible. Patients with psychotic symptoms will be included and will not be stratified. Patients with co-morbid conditions are allowed to participate due to the study clinical reality approach ("more likely naturalistic study").

The patient or his/her legal representative should understand the nature of the study and sign the Informed Consent.

Eligible patients under pharmacological treatments proceed to a wash-out period according to the medication in use: 1 week for antidepressants (except fluoxetine and irreversible MAOI), antipsychotics (except clozapine), lithium, valproate, carbamazepine and other anticonvulsants; 2 weeks for irreversible MAOI; 4 weeks for fluoxetine and clozapine.

### Sample Size and Randomization

Sample size was calculated comparing the mean difference in CGI-BP-M scores between baseline and endpoint for the two groups. If we compare this using a Student's t test we find that with 50 patients (25 in each group) we achieve 80% power (in a 5% of significance level test) to detect a difference of 0.8 standard deviation, which can be considered a large effect size [11]. Due to the exploratory nature of this study and the difficulties of compliance in a long term trial, this seems to be an acceptable value.

The random allocation sequence was computer generated by a biostatistician. Patients were enrolled by clinicians who have their code revealed by the research monitor when assigned to interventions. Blinding the outcome assessor is done keeping their evaluation independent to the clinician.

### Study phases

Patients will be followed up for 8 weeks in phase I (acute treatment), 6 months in phase II (continuation treatment) and 12 months in phase III (maintenance treatment). Scales raters will be blind to the treatment. During phase II and III will continue only patients that achieve response, measured according to initial symptoms score in phase I. Patients with a new episode of any polarity detected in two consecutives visits will be discontinued from the study.

The definition of clinical course is defined according to Hirschfeld et al (2007) [12], Bauer et al (2007) [13] and Tohen et al (2009) [14]:

Non response: reduction ≤ 25% in severity of symptoms;

Partial response: improvement of symptoms between 26 and 49%;

Response: reduction ≥ 50% in severity of symptoms;

Remission: minimal or no symptoms for at least 1 week;

Sustained remission: at least 8 weeks of remission;

Relapse/recurrence: return of the criteria for the syndrome;

Roughening: return of symptoms in a subsyndromal level.

### Primary outcomes

Primary outcome will be the evaluation of changes in mean scores on CGI-BP-M between baseline and endpoint at the end of each phase of the study.

### Secondary outcomes

Secondary outcome will include the proportion of patients that achieve remission and response to each treatment at the end of each phase of the study, according to improvement in rating scales (HAM-D; MADRS; YMRS) and in clinical global impression specific for BD (CGI-BP-M) [15]. The CGI-BP-M is a clinician rating scale modified for Bipolar Disorder concerning to treatment response and consists of 3 sub-scales evaluating the severity of mania, depression and the whole disease symptoms.

Other outcome parameters are: safety and tolerability; quality of life and social adjustment; and cognitive impairment. These parameters will be evaluated comparing the endpoint measures of each phase with baseline. Safety and tolerability: according to the clinical evaluation of adverse effects and the measure on the UKU side effect rating scale [16]. UKU consists of 48 items clinician rated evaluating side effects in psychic, neurologic, autonomic and other domains. Quality of life and social adjustment: measure with the WHOQoL-BREF [17] and Social Adjustment Scale [18]. These instruments were translated and validated to Portuguese [19,20]. WHOQOL-BREF is a self report 26 items scale comprising 4 domains: physical, psychological, social and environment. The Social Adjustment Scale is a self report 54 questions instrument that measures instrumental and expressive role performance over the past two weeks. Cognitive impairment: neuropsychological tests (Wisconsin Card Sorting Test [21], Stroop Color Word Test [21], WASI - Wechsler Abbreviated Scale of Intelligence [22], Trail Making Test [21] and others), which are available in Portuguese. All investigators received appropriate training, and inter-rater reliability is periodically assessed.

### Planned Analyses

The primary analysis will be the evaluation of the mean scores on CGI-BP-M between baseline and endpoint in both groups. We will also evaluate differences comparing the two groups of treatment concerning total number of patients in full remission at the end of the study and the reason and time to drop out. Continuous data will be represented by mean and standard deviation (SD). Categorical variables will be described by table of frequencies. The results of all statistical comparisons of the treatment groups will be presented as a 2-sided p values rounded to 3 decimal places. The criterion for statistical significance in all comparisons will be p ≤ 0.05.

Continuous variables will be compared using repeated measures analysis of variance (ANOVA), with group of treatment and study phase as factors. Rates of response, remission and drop-outs will be compared between the two groups using a Pearson's X^2 ^test for categorical data. Dichotomous measures will also be compared using odds ratios and 95% confidence limits.

The correlation of clinical issues, quality of life, social adjustment and cognitive impairment will be evaluated by Pearson's correlation.

## Results

### Current Status of LICAVAL

LICAVAL is currently in progress, with patients in phase I or II. It will extend during the next two years.

## Discussion

Trials comparing specific treatments efficacy in BD (head to head) can show relevant information in clinical practice.

Usual limitations in clinical trials are:

- Specific clinical forms of BD (Mania/Mixed or Depression) for each treatment tested.

- There is little information about how the treatment in acute phase should progress to maintenance or which factors from the acute treatment could predict recurrences during maintenance treatment.

- The heterogeneity of patients, considering course of illness (chronic versus non chronic), in clinical trials can shadow important treatment implications for specific populations (younger versus older, for example).

## Conclusions

Due to the substantial increase in treatment options, guidelines and algorithms are used in an effort to enhance the cost-effectiveness of care by reducing the number of treatment options [13]. Although limitations can be addressed in the use of these algorithms, they represent the available data concerning the levels of evidence of each treatment option.

Lithium, anticonvulsants and atypical antipsychotics appear in all guidelines with different recommended use according to clinical presentation, showing different levels of efficacy. Lithium, Valproate and Carbamazepine have strong evidence of benefits in BD treatment, although each of them have its particularities. Evidence based knowledge concerning maintenance treatment with combination treatment are still needed in clinical practice.

## Competing interests

RAM has acted as a consultant to and conducted research sponsored by companies with developments in the area of bipolar and depressive disorders (Servier, Bristol Myers Squibb, Solvay Pharma, Abbott, Astra Zêneca, Pfizer, and Roche). FND has acted as a consultant to and conducted research sponsored by companies with developments in the area of bipolar and depressive disorders (Abbott, Novartis **e **Eurofarma). DHM was a speaker and member of the board of Abbott and Astra-Zeneca. RNC, LFC, DSB, MGSS and CRLG declare no competing interests.

## Authors' contributions

RAM has made substantial contribution to conception and study design and has been involved in revising the manuscript for important intellectual content. RNC, LFC, DSB, MGSS, CRLG, FND and DHM contributed to conception and study design and clinical assistance. MGSS contribute as blind rater. All authors read and approved the final manuscript.

## References

[B1] Goldberg JF Optimizing treatment outcomes in bipolar disorder under ordinary conditions J Clin Psychiatry 2008 69 Suppl 3 11 9 18533757

[B2] American Psychiatric Association Diagnostic and Statistical Manual of Mental Disorders Text Revision 2000 Fourth Washington DC, American Psychiatric Association

[B3] Beyer JL An evidence-based medicine strategy for achieving remission in bipolar disorder J Clin Psychiatry 2008 69 Suppl 3 31 7 18533760

[B4] Perlis RH Ostacher MJ Patel JK Marangell LB Zhang H Wisniewski SR Ketter TA Miklowitz DJ Otto MW Gyulai L Reilly-Harrington NA Nierenberg AA Sachs GS Thase ME Predictors of recurrence in bipolar disorder: primary outcomes from the Systematic Treatment Enhancement Program for Bipolar Disorder (STEP-BD) Am J Psychiatry 2006 163 2 217 24 10.1176/appi.ajp.163.2.217 16449474

[B5] Goodwin FK Rationale for long-term treatment of bipolar disorder and evidence for long-term lithium treatment J Clin Psychiatry 2002 63 Suppl 10 5 12 12392347

[B6] Gershon S Chengappa KN Malhi GS Lithium specificity in bipolar illness: a classic agent for the classic disorder Bipolar Disord 2009 11 Suppl 2 34 44 10.1111/j.1399-5618.2009.00709.x 19538684

[B7] Pérez-Ceballos MA Vega-Gil N Sánchez MB Armijo JA Use of antiepileptic drugs in bipolar disorder Actas Esp Psiquiatr 2006 34 1 55 64 16525906

[B8] Bowden CL Anticonvulsants in bipolar disorders: current research and practice and future directions Bipolar Disord 2009 11 Suppl 2 20 33 10.1111/j.1399-5618.2009.00708.x 19538683

[B9] Weisler RH Cutler AJ Ballenger JC Post RM Ketter TA The use of antiepileptic drugs in bipolar disorders: a review based on evidence from controlled trials CNS Spectr 2006 11 10 788 99 17008822 10.1017/s1092852900014917

[B10] Schulz KF Altman DG Moher D Group C CONSORT 2010 Statement: updated guidelines for reporting parallel group randomised trials BMC Med 2010 8 1 18 10.1186/1741-7015-8-18 20334633 PMC2860339

[B11] Cohen J Statistical power analysis for the behavior sciences 1988 2 Hillsdale, NJ: Erlbaum

[B12] Hirschfeld RMA Calabrese JR Frye MA Lavori PW Sachs G Tase M Wagner KD Defining the clinical course of bipolar disorder: response, remission, relapse, recurrence, and roughening Psychopharmacol Bull 2007 40 3 7 14 18007564

[B13] Bauer M Alda M Priller J Young LT International Group For The Study Of Lithium Treated Patients (IGSLI) Implications of the neuroprotective effects of lithium for the treatment of bipolar and neurodegenerative disorders Pharmacopsychiatry 2003 36 Suppl 3 S250 4 14677087 10.1055/s-2003-45138

[B14] Tohen M Frank E Bowden CL Colom F Ghaemi SN Yatham LN Malhi GS Calabrese JR Nolen WA Vieta E Kapczinski F Goodwin GM Suppes T Sachs GS Chengappa KR Grunze H Mitchell PB Kanba S Berk M The International Society for Bipolar Disorders (ISBD) Task Force report on the nomenclature of course and outcome in bipolar disorders Bipolar Disord 2009 11 5 453 73 10.1111/j.1399-5618.2009.00726.x 19624385

[B15] Vieta PE Torrent FC Martínez-Arán A Colom VF Reinares GM Benabarre HA Comes FM Goikolea AJM A user-friendly scale for the short and long term outcome of bipolar disorder: the CGI-BP-M Actas Esp Psiquiatr 2002 30 5 301 4 12372226

[B16] Lingjaerde O Ahlfors UG Bech P Dencker SJ Elgen K The UKU side effect rating scale. A new comprehensive rating scale for psychotropic drugs and a cross-sectional study of side effects in neuroleptic-treated patients Acta Psychiatr Scand Suppl 1987 334 1 100 10.1111/j.1600-0447.1987.tb10566.x 2887090

[B17] The WHOQOL Group What Quality of Life? 1996 World Health Forum. WHO: Geneva 354 56 9060228

[B18] Weissman MM Bothwell S Assessment of social adjustment by patient self-report Arch Gen Psychiatry 1976 33 1111 5 962494 10.1001/archpsyc.1976.01770090101010

[B19] Fleck MPA O instrumento de avaliação de qualidade de vida da Organização Mundial da Saúde (WHOQOL-100): características e perspectivas Ciênc. Saúde Coletiva 2000 5 1 33 38 10.1590/S1413-81232000000100004

[B20] Gorenstein C Andrade L Moreno RA Bernik MA Nicastri S Cordás TA Camargo AP Gorenstein C, Andrade LHSG, Zuardi AW Escala de auto-avaliação de adequação social: validação da versão em língua portuguesa Escalas de avaliação clínica em psiquiatria e psicofarmacologia. São Paulo: Lemos 2000 401 414

[B21] Spreen O Strauss E A Compendium of neuropsychological tests 1998 Oxford: Oxford University Press

[B22] Wechsler D Wechsler Abbreviated Scale of Inteligence 1999 New York: Psychological Corporation

